# Alignment of Synaptic Vesicle Macromolecules with the Macromolecules in Active Zone Material that Direct Vesicle Docking

**DOI:** 10.1371/journal.pone.0069410

**Published:** 2013-07-22

**Authors:** Mark L. Harlow, Joseph A. Szule, Jing Xu, Jae Hoon Jung, Robert M. Marshall, Uel J. McMahan

**Affiliations:** 1 Department of Neurobiology, Stanford University School of Medicine, Stanford, California, United States of America; 2 Department of Applied Physics, Stanford University School of Humanities and Sciences, Stanford, California, United States of America; 3 Department of Physics, Stanford University School of Humanities and Sciences, Stanford, California, United States of America; 4 Department of Biology, Texas A&M University, College Station, Texas, United States of America; Virginia Tech Carilion Research Institute, United States of America

## Abstract

Synaptic vesicles dock at active zones on the presynaptic plasma membrane of a neuron’s axon terminals as a precondition for fusing with the membrane and releasing their neurotransmitter to mediate synaptic impulse transmission. Typically, docked vesicles are next to aggregates of plasma membrane-bound macromolecules called active zone material (AZM). Electron tomography on tissue sections from fixed and stained axon terminals of active and resting frog neuromuscular junctions has led to the conclusion that undocked vesicles are directed to and held at the docking sites by the successive formation of stable connections between vesicle membrane proteins and proteins in different classes of AZM macromolecules. Using the same nanometer scale 3D imaging technology on appropriately stained frog neuromuscular junctions, we found that ∼10% of a vesicle’s luminal volume is occupied by a radial assembly of elongate macromolecules attached by narrow projections, nubs, to the vesicle membrane at ∼25 sites. The assembly’s chiral, bilateral shape is nearly the same vesicle to vesicle, and nubs, at their sites of connection to the vesicle membrane, are linked to macromolecules that span the membrane. For docked vesicles, the orientation of the assembly’s shape relative to the AZM and the presynaptic membrane is the same vesicle to vesicle, whereas for undocked vesicles it is not. The connection sites of most nubs on the membrane of docked vesicles are paired with the connection sites of the different classes of AZM macromolecules that regulate docking, and the membrane spanning macromolecules linked to these nubs are also attached to the AZM macromolecules. We conclude that the luminal assembly of macromolecules anchors in a particular arrangement vesicle membrane macromolecules, which contain the proteins that connect the vesicles to AZM macromolecules during docking. Undocked vesicles must move in a way that aligns this arrangement with the AZM macromolecules for docking to proceed.

## Introduction

Initial events in the synaptic transmission of nerve impulses at chemical synapses occur at active zones on the plasma membrane of axon terminals of the presynaptic neuron [1]–[4]. The arrival of an impulse at an active zone causes calcium channels concentrated in the presynaptic plasma membrane to open. The influx of calcium into the cytosol triggers protein-mediated fusion of the membrane of synaptic vesicles with the presynaptic membrane and release of the vesicles’ neurotransmitter into the synaptic cleft to act on the postsynaptic cell. Prior to these events, the vesicles move toward and become held at the presynaptic membrane by a process known as docking. Biochemistry has shown that docking involves the interaction of proteins of the vesicle membrane with stable proteins of the active zone [5]. Such interactions include those formed by, for example, the cytosolic portion of the vesicle membrane proteins synaptobrevin with the cytosolic portion of the presynaptic membrane proteins syntaxin and SNAP-25 [6], [7]. The results of electron tomography on the structure and function of the simply arranged active zones of axon terminals at neuromuscular junctions (NMJ’s) indicate that most, if not all, proteins involved in vesicle docking contribute to the common active zone organelle, active zone material (AZM) [8]–[11]. The structure of AZM and the manner in which vesicles connect to it during docking provide particular insights about the sequence of steps involved in the process.

AZM is a dense aggregate of macromolecules. As viewed in the 2-dimensional (2D) images obtained by conventional transmission electron microscopy on sections from fixed and stained tissue, it is attached to the cytoplasmic surface of the presynaptic membrane [3], [11], [12]. It extends several tens of nanometers vertical to the membrane into the cytoplasm. Its shape and distribution within an active zone vary among animal species and from one neuron type to another within a species [13]. Nevertheless, the AZM is typically next to synaptic vesicles docked on the presynaptic membrane. Because AZM macromolecules are much thinner than the thinnest tissue sections that can be cut (∼30 nm) and overlap each other in a section’s depth axis, they are difficult to study by conventional electron microscopy. However, they can be examined in considerable detail by electron tomography. Electron tomography relies on multiple 2D transmission electron microscope images of a specimen collected at different tilt angles to generate a 3-dimensional (3D) reconstruction of the specimen [14]. For such reconstructions of sections containing active zones, serial virtual slices thinner than AZM macromolecules are made through the volume. The AZM macromolecules can, then, be studied in 3D either alone or together with other structures by using the serial slices for segmenting them from the reconstructed volume and generating surface models of them (e.g. [8]–[11], [15]–[18]).

Electron tomography on the active zones of frog NMJ’s routinely fixed and stained with glutaraldehyde, osmium tetroxide and uranyl acetate at room temperature has shown that the AZM’s macromolecules are elongate, and that most of them are constituents of a highly ordered network [8], [11]. The macromolecules fall into several logically distinct classes based on their position, shape, dimensions, and the connections they form. Each macromolecule within a class appears large enough in diameter to accommodate several proteins, and, although some proteins may extend from one macromolecule into another at their connection sites, each class is considered functionally unique. In axon terminals fixed at rest, multiple members of four classes of AZM macromolecules are connected to each vesicle docked on the presynaptic membrane. Some or all may contain the cytosolic portions of presynaptic membrane proteins and vesicle membrane proteins known to interact during vesicle docking on and fusion with the presynaptic membrane. Three of the four classes are situated at different distances from the presynaptic membrane, and the connections each class forms with a vesicle are confined to a specific domain on the vesicle’s surface. In activated axon terminals, fixed during replacement of docked vesicles by previously undocked vesicles, undocked vesicles near vacated docking sites on the presynaptic membrane are connected to the same classes of AZM macromolecules connected to docked vesicles in resting terminals. The number of classes and the total number of AZM macromolecules to which the undocked vesicles are connected are inversely proportional to the vesicles’ distance from the presynaptic membrane. These findings have led to the conclusion that AZM directs undocked vesicles toward docking sites on the presynaptic membrane by forming a succession of stable macromolecular interactions with protein components of the vesicle membrane, and that these same interactions persist to help hold docked vesicles in contact with the presynaptic membrane. They have also led to questions of how the vesicle proteins involved in docking are distributed in the vesicle membrane and how these proteins come to associate with the different classes of AZM macromolecules during docking [11].

Although the staining of cellular structures by osmium tetroxide and uranyl acetate can vary depending on whether the staining is done in aqueous media at room temperature or in acetone at low temperature by freeze-substitution [19], at frog NMJ’s the staining of AZM macromolecules, of the membrane of synaptic vesicles and of the presynaptic membrane is generally the same regardless of which one of the methods is used [11]. However, we observed, while using such methods, that staining with osmium tetroxide and uranyl acetate in acetone by freeze-substitution exposed an interconnected assembly of macromolecules in the lumen of synaptic vesicles not evident after staining at room temperature. Biochemical analyses of the protein composition of synaptic vesicles make it likely that the intraluminal macromolecules are composed of the luminal portions of proteins in the vesicle membrane [20]. Membrane proteins with luminal portions include those that have cytosolic portions known to interact with active zone proteins during docking [5]. In this study, we characterized by electron tomography the arrangement and associations of the luminal assemblies at frog NMJ’s. Our results provide evidence that they anchor macromolecules in the vesicle membrane in a particular arrangement, and certain of these macromolecules contain proteins that connect to proteins in the different classes of AZM macromolecules during docking.

## Results

### Background

**Figure 1:**
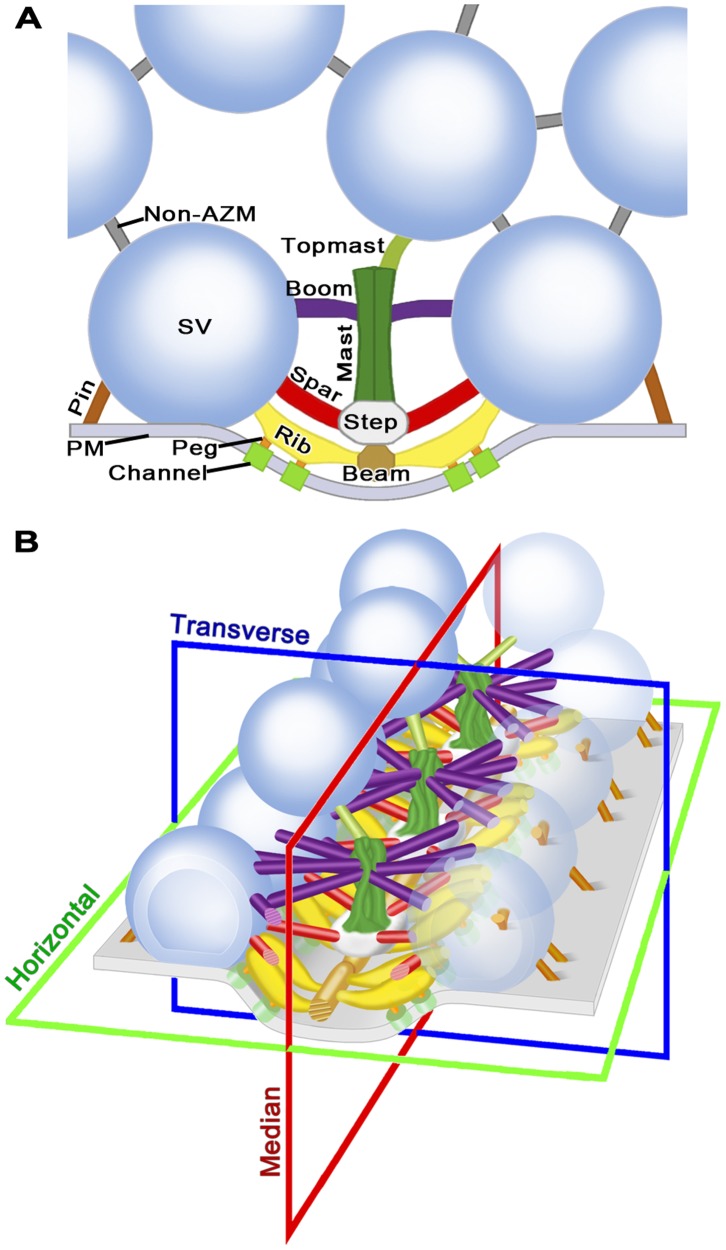
Layout of the active zone at the frog’s NMJ. (A) Composite diagram viewed in the active zone’s transverse plane. The main body of the AZM is between the two synaptic vesicles (SV) docked on the presynaptic membrane (PM). At the core of the main body are the beam, step and mast, each connected to the docked vesicles by the horizontally arranged ribs, spars and booms, respectively. Pegs, which were not included in this study (but see [8]–[10]) connect the ribs to channels in the presynaptic membrane, while pins, which are AZM macromolecules away from the AZM’s main body connect the docked vesicles directly to the presynaptic membrane. A topmast links the mast to an undocked vesicle. Non-AZM macromolecules connect the docked vesicles to nearby undocked vesicles and are similar in appearance to macromolecules linking undocked vesicles to each other. (B) Schematic of a short segment of the active zone showing the 3D relationship of AZM macromolecules to docked vesicles and those undocked vesicles linked to topmasts, with indicators of the active zone’s horizontal, median and transverse planes. The color code for the structures shown here is the same for all Figures in this report. A complete description of the organization of AZM and non-AZM macromolecules can be found in [11].

The main body of the AZM at each active zone of the frog’s NMJ is in the form of a narrow band that extends along the axon terminals’ presynaptic plasma membrane for, in many cases, a micrometer or more (Figure 1; see also [8], [11], [12], [21]). It is attached to a shallow evagination of the presynaptic membrane, the active zone ridge, throughout its length, is ∼50 nm wide, and, at regular intervals, extends ∼75 nm from the presynaptic membrane into the cytoplasm. In resting terminals, docked synaptic vesicles are in a row along each side of the main body of the AZM, while numerous undocked vesicles are distributed with no apparent order in a cloud lateral and deep to the active zone. The outer diameter of the docked vesicles, as determined in 3D reconstructions used in this study, was ∼51 nm (51.3±3.3 nm; n = 12 docked vesicles from four reconstructions), which is consistent with our previous measurements [10], [11]. The luminal diameter of the same docked vesicles was ∼36 nm (35.7±2.6 nm). We refer below to the three cardinal planes of the active zone (Figure 1B): the horizontal plane, which is parallel to the presynaptic membrane beyond the active zone ridge; the median plane, which is orthogonal to the horizontal plane and parallel to the long axis of the main body of the AZM; and the transverse plane, which is orthogonal to both the horizontal plane and the median plane.

The four classes of AZM macromolecules connected to docked vesicles are called ribs, pins, spars and booms (Figure 1A). Ribs, spars and booms are components of the main body of AZM. Each of these classes arises from a different core AZM macromolecule in the main body - beams, steps and masts, respectively - and extends nearly parallel to the presynaptic membrane to connect to the membrane of docked vesicles at a different distance from the presynaptic membrane; ribs are closest to the presynaptic membrane, booms the furthest. Pins, which are outside the AZM’s main body, arise from the presynaptic membrane and extend almost vertically to connect to the vesicles on the hemisphere that faces away from the main body of AZM. The connection sites of the ribs and pins on the vesicles are distributed around the fusion domain, i.e., the area of the vesicle membrane in direct contact with the presynaptic membrane [11]. Each docked vesicle is connected on average to 4 ribs, 4 pins, 2 spars and 5 booms (Figure 1) [8], [9], [11]. There are also several non-AZM macromolecules having similar dimensions to those of the AZM. They are connected primarily to the hemisphere of each docked vesicle that faces away from the main body of the AZM (Figure 1A), and their positioning is distinct from that of pins. They link docked vesicles to nearby undocked vesicles and other organelles, and, thus, resemble macromolecules that connect undocked vesicles to each other and other organelles throughout the axon terminal [22], [23]. Although the connections of such macromolecules to docked and undocked vesicles must regulate vesicle trafficking in general [24], [25], there is no evidence, to date, that they specifically direct undocked vesicles to docking sites on the presynaptic membrane as do the connections formed between undocked vesicles and AZM macromolecules. Extending from the deep end of a mast are one to three elongate macromolecules called topmasts, each of which is connected to an undocked vesicle. We have suggested that, because of the constancy of their close proximity to docked vesicles, the undocked vesicles connected to topmasts preferentially replace docked vesicles when they fuse with the presynaptic membrane [11]. Here we examine the relationship of the luminal assembly of macromolecules in docked vesicles to the connections of AZM and non-AZM macromolecules on the external surface of the vesicle membrane.

It is difficult to study the lumen of synaptic vesicles, such as those at the frog’s NMJ, when they are imaged in tissue sections by conventional 2D electron microscopy [3], [11], [26], [27]. Many of the vesicles in tissue sections thin enough for adequate spatial resolution are not included in their entirety, and the extent to which any such vesicle is included is often uncertain. Moreover, cytoplasmic structures superficial and/or deep to a vesicle in the section are projected in the same image plane as the lumen. The serial virtual slices through the reconstructed volumes of tissue sections we used for this electron tomography study were a small fraction of the diameter of a vesicle, making it possible to determine the extent to which a vesicle was included in a section and to systematically search for luminal structures.

**Figure 2:**
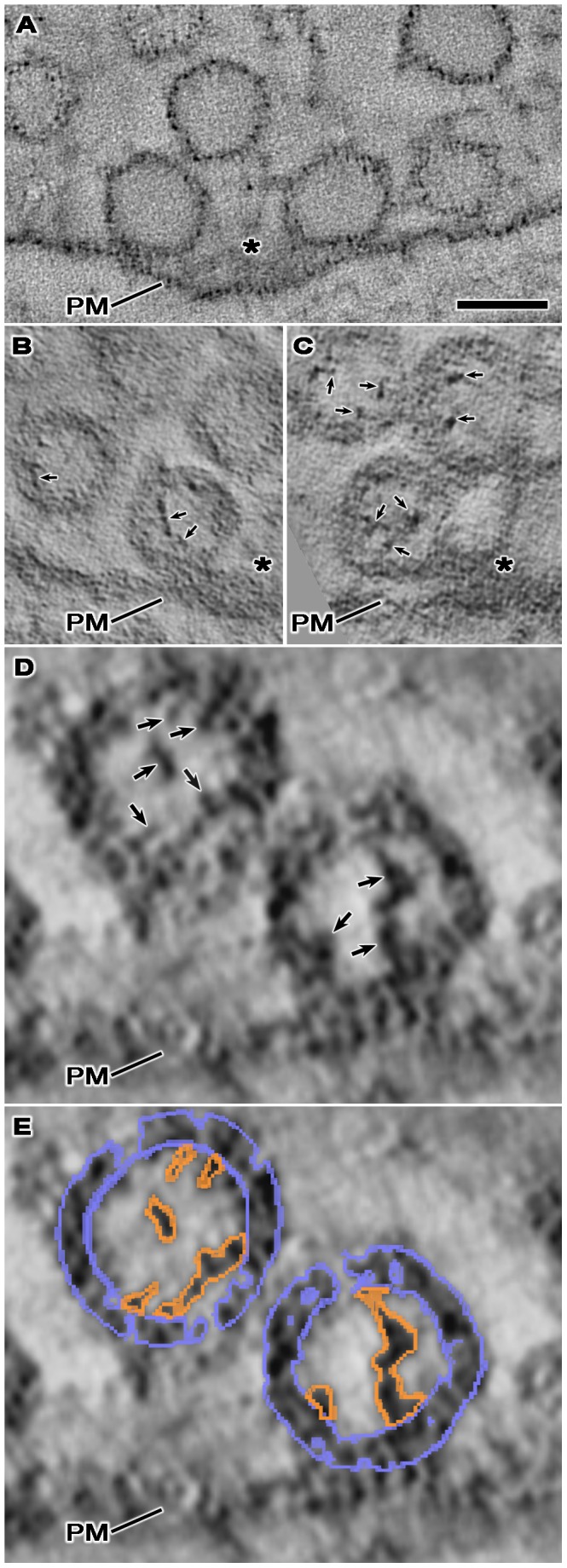
The lumen of synaptic vesicles after staining under different conditions. Each panel shows a virtual slice, 1–2 nm thick, through vesicles at or near an active zone (PM, presynaptic membrane; asterisk, main body of AZM). (A) The NMJ was fixed and stained with glutaraldehyde, osmium tetroxide and uranyl acetate at room temperature. (B) The NMJ was fixed with glutaraldehyde at room temperature and, after rapid freezing, fixed further and stained with osmium tetroxide and uranyl acetate in acetone by freeze-substitution. (C–E) The NMJ’s were fixed by rapid freezing, stained with osmium tetroxide and uranyl acetate in acetone by freeze-substitution. While the lumen of synaptic vesicles at the NMJ fixed and stained with glutaraldehyde, osmium tetroxide and uranyl acetate at room temperature appears empty (A), the lumen of synaptic vesicles at the NMJ’s, stained with osmium tetroxide and uranyl acetate in acetone by freeze-substitution, regardless of whether they were fixed by rapid freezing or with glutaraldehyde at room temperature, contains an assembly of macromolecules (B–D; arrows). The staining of the vesicle membrane and luminal assembly in all cases is particulate. The particles in the luminal assemblies have greater electron density than those in the membrane. In (E), the vesicle membrane and luminal assemblies shown in (D) are overlaid with the portion of the 3D surface models of the entire vesicle membrane (blue) and the luminal assembly (orange) that were generated from this virtual slice, which establishes the limits of the membrane width throughout the membrane’s circumference, the edges of the luminal assemblies, and the sites of connection of the luminal assemblies to the membrane. Scale bar (A–C) = 50 nm, (D–E) = 25 nm.

We found no discernable luminal structures in serial virtual slices through each of the more than 100 whole vesicles examined in reconstructed tissue sections from muscles fixed and stained with glutaraldehyde, osmium tetroxide and uranyl acetate at room temperature (Figure 2A). Serial slices through reconstructed vesicles from muscles fixed and stained at room temperature by osmium tetroxide without pretreatment with glutaraldehyde also did not reveal luminal structures (data not shown). On the other hand, we observed an assembly of elongate macromolecules connected to the luminal surface of vesicle membranes in serial virtual slices through each of the more than 75 whole vesicles examined in sections from tissue fixed by rapid freezing and stained with osmium tetroxide and uranyl acetate in acetone by freeze-substitution (Figure 2C–E, 3A–E), a method that also exposes AZM macromolecules and the other structures seen by the routine staining methods used at room temperature. The luminal assemblies were also evident in synaptic vesicles in muscles fixed with glutaraldehyde at room temperature and stained with osmium tetroxide and uranyl acetate in acetone by freeze-substitution (Figure 2B), but the signal to noise ratio for the assemblies was generally less than for the luminal assemblies of vesicles in muscles fixed by rapid freezing [19]. In all cases, the heavy metal staining of structures was particulate. However, in muscles stained by freeze-substitution the frequency and electron density of particles was generally greater for the luminal assemblies than for the vesicle membrane (Figure 2B–D). Due to the non-uniform frequency of particles of stain in the vesicle membrane, the *z*-axis curvature of the membrane and the thinness of the serial virtual slices, there were often small gaps in the membrane’s outlines in any particular slice. Our method of segmentation for generating surface models [9], which is based on gray-scale levels in 3D, provided a way to establish a membrane’s luminal and outer surface throughout each slice (Figure 2D,E) and made it possible to map the connection sites of the luminal assembly on the luminal surface, as we have done here and previously for the connection sites of AZM macromolecules on the outer surface [8], [9], [11].

**Figure 3:**
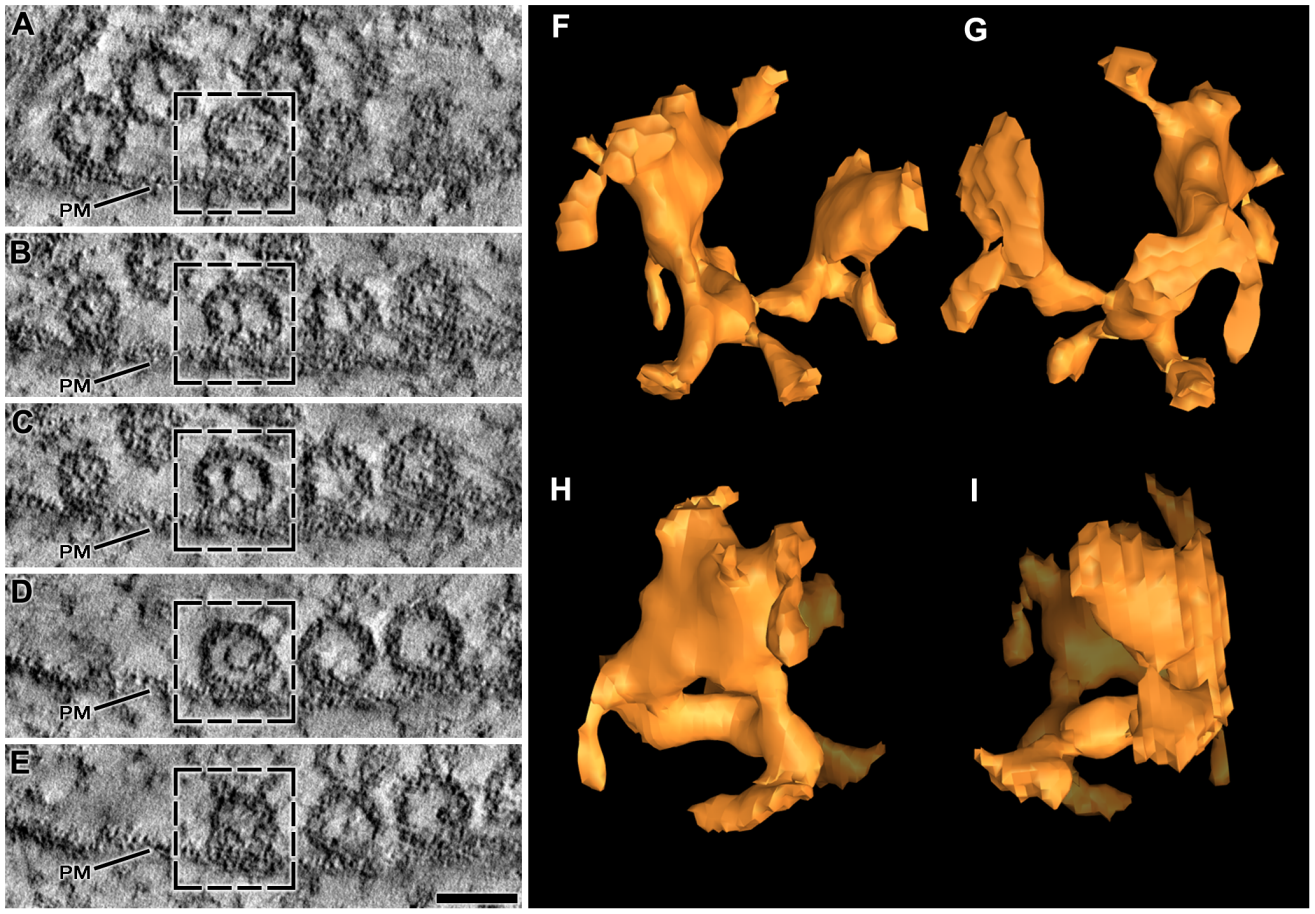
Shape of the luminal assembly of macromolecules in the principal vesicle. (A–E) Selected 1 nm thick virtual slices from a series made through one of the primary reconstructions. The section from which the reconstruction was made was cut in the active zone’s median plane; the virtual slices are shown in the same plane. Four vesicles are docked in a row on the presynaptic membrane (PM). Undocked vesicles are nearby. All of the vesicles contain luminal assemblies of macromolecules. The box in each virtual slice outlines the so-called principal vesicle. (F–I) 3D surface model of the principal vesicle’s luminal assembly shown in different degrees of rotation. In (F) the assembly is viewed from the median plane of the AZM with the portion facing the presynaptic membrane downward. In (G) the assembly is rotated 180 degrees around the vertical axis of its orientation in (F). In (H) and (I) the assembly is rotated 90 degrees to the right and left, respectively, around the vertical axis of the orientation in (F). The assembly has a bilateral arrangement of four irregular arms, which radiate from below and behind the center of the vesicle. Nubs of varying lengths arise from the arms. Scale bar (A–E) = 50 nm.

We started by studying in detail the assemblies of luminal macromolecules and their relationships in reconstructions of two sections, each containing an active zone from different axon terminals in the same muscle. The muscle was fixed by rapid freezing and stained by the freeze-substitution method. We refer to these reconstructions as the *primary reconstructions*. They were among the first in which we detected luminal assemblies. We, then, compared specific results from the primary reconstructions to those from other reconstructions in the same and four different muscles, each from a different frog. Two of these muscles had also been fixed by rapid freezing and stained with osmium tetroxide and uranyl acetate in acetone by freeze-substitution while the other two had been fixed with glutaraldehyde at room temperature before staining with osmium tetroxide and uranyl acetate in acetone by freeze-substitution. The arrangement of macromolecules reported here was the same regardless of the method used for fixation.

### The Shape of the Assembly and the Amount of the Luminal Volume it Occupies

**Figure 4:**
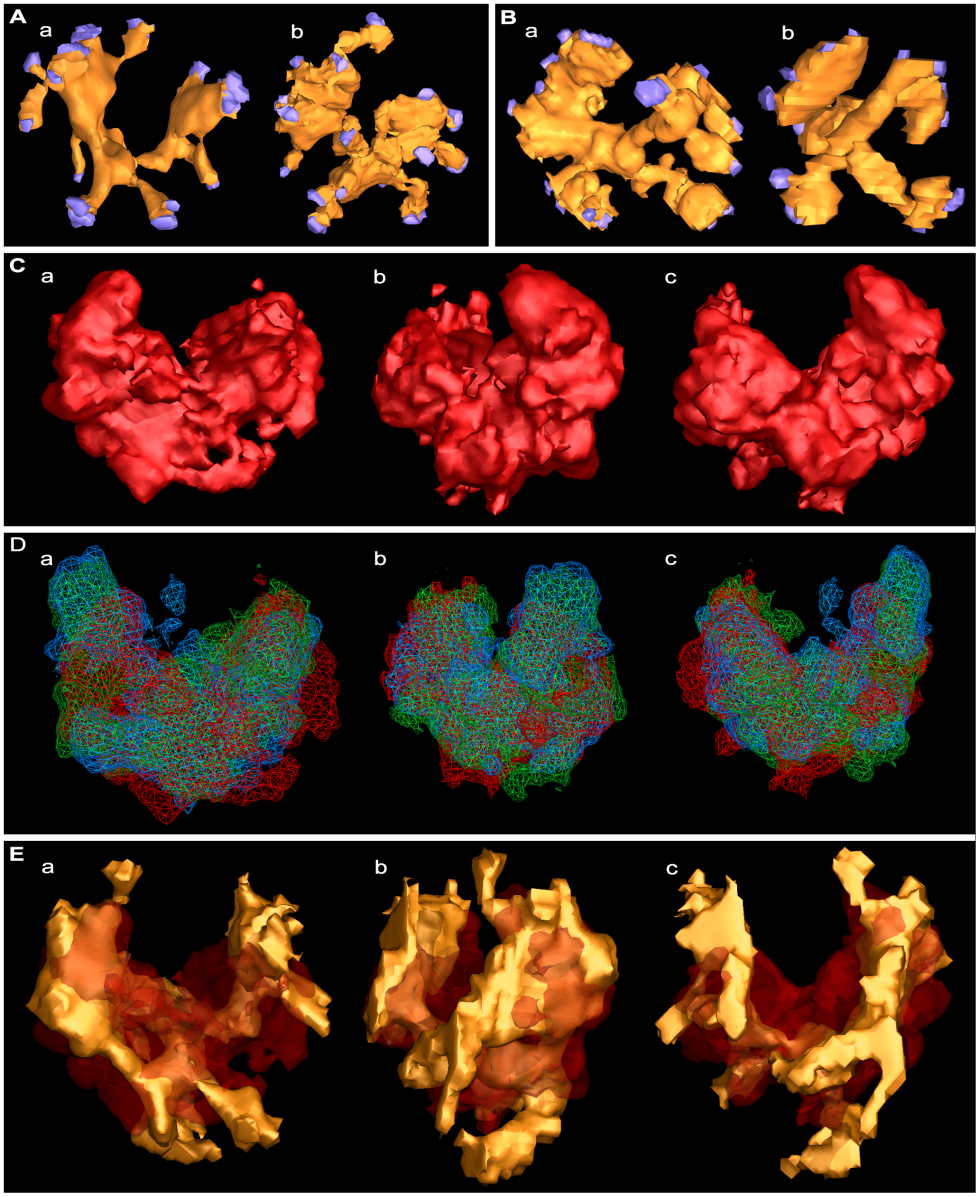
Similarity in the shape of the luminal assemblies and their association with the vesicle membrane. (Aa) Surface model of the assembly from the principal docked vesicle shown in Figure 3F–I. (Ab) Surface model of an assembly from another docked vesicle. (Ba,b) Surface models of assemblies from undocked vesicles. The assemblies in (Ab,Ba,Bb) were rotated until their shape matched that of the principal vesicle. The NMJ’s used for the assemblies in (A) and (B) were fixed by rapid freezing. In all cases, the assemblies are bilateral with irregular arms radiating from near the center of the vesicle. Nubs arise from the arms to connect at their end to the vesicle membrane; the terminal 3 voxels of each nub were made blue to mark its connection site on the membrane. (Ca–c) Alignment model generated by gray-scale density alignment of the 12 docked and undocked vesicles in the two primary reconstructions, which included those shown in (Aa,Ba). In (Ca) the alignment model is oriented according to the orientation of the surface model of the assembly from the principal vesicle as shown in (Aa). In (Cb) the alignment model is rotated 90 degrees to the right around the vertical axis in (Ca). In (Cc) the alignment model is rotated 180 degrees around the vertical axis in (Ca). The alignment model shows a bilateral arrangement of 4 radiating arms as do the models of individual assemblies in (Aa,b,Ba,b). (Da–c) The alignment model (red) generated from gray-scale density alignment of the 12 luminal assemblies in (Ca–c) aligned together with two alignment models (blue and green) generated from the same 12 assemblies represented according to their surface models. The different orientations of the superimposed alignment models are the same as for that in (Ca–c). The alignment models, which were calculated to have 95% 3D overlap, were used to measure the similarity in the shape of the assemblies from vesicle to vesicle and for establishing the orientation of the shape from vesicle to vesicle. (Ea–c) The surface model in (Aa) inserted into the alignment model in (Ca–c) according to its position in the alignment model. The different orientations of the combined models are the same as in (Ca–c). The portions of the surface model least included in the alignment model are the nubs, probably due to the nubs being smaller and/or having a somewhat greater variability in positioning than the arms among the surface models used for generating the alignment model.

To examine the shape of the luminal assembly of macromolecules in individual vesicles, we began by generating surface models of the assembly, the vesicle membrane and other components of the active zone in a way that maintained their relative positions. This made it possible to view the assemblies alone or together with the other active zone structures at any degree of rotation, and details of their shape could be related to the cardinal planes of the active zone (shown in Figure 1B). We started with a docked vesicle in one of the primary reconstructions because the signal to noise ratio of its luminal assembly was especially high when viewed in serial slices (Figures 3A–E). We refer to it below as the *principal vesicle* in comparing the shape and orientation of its luminal assembly to that of the luminal assemblies in other vesicles. Four conjoined elongate macromolecules, arms, radiated from a focal point near the center of its lumen (Figures 3F–I). Each arm was larger near the vesicle’s membrane than it was near the vesicle’s center and it had an irregular topography. When it was viewed from the median plane of the active zone, with the portion facing the vesicle’s fusion domain downward, a different arm extended into each of the four quadrants of the vesicle. The focal point was below and behind the center of the vesicle, which conferred geometric chirality to the bilateral assembly. The two arms in the quadrants that included the membrane’s fusion domain were shorter than those in the quadrants away from the fusion domain. Several relatively thin macromolecules of various lengths, called nubs, extended from each of the arms to connect to nearby regions of the vesicle membrane (Figure 4Aa).

The shapes of the luminal assemblies in other docked vesicles and in undocked vesicles, when represented in surface models, were strikingly similar to that of the principal vesicle; a similar number of arms in the same relative positions radiated from a focal point near the vesicle center, and they were connected to the vesicle membrane by nubs (Figures 4A,B). There were about 25 nub connection sites on the membranes of both docked and undocked vesicles (docked, 23.8±4.4 SD, n = 10; undocked, 24.8±2.6 SD, n = 5; not significantly different, p>0.65 as determined by *t*-test). In a few instances, arms bifurcated and reunited along their length, and nubs bifurcated before terminating on the vesicle membrane (not shown). Moreover, in certain assemblies there were discontinuities at various points along the length of some arms and between a few nubs and arms. Such discontinuities may well have resulted from continuous structures having failed to stain continuously. For each of the 12 vesicles of the primary reconstructions, we determined the fraction of the luminal voxels that were confined to the stained assembly; the results showed the luminal assemblies occupied 10±1% (range) of the luminal volume.

To test for the similarity in the overall configuration of the luminal assembly of macromolecules from vesicle to vesicle we aligned by rotation and translation the individual assemblies in the 12 vesicles of the primary reconstructions (Figure 4C,D). We generated three alignment models, each using a reference-free, dual-phase algorithm [28]. Each method had unique advantages and disadvantages (see Methods). For generating one alignment model, the assemblies from each vesicle were represented according to their gray-scale voxel densities; for generating the other two alignment models the assemblies from each vesicle were represented as surface models. The alignment models generated using the different representations were very similar (Figure 4D); the volume of each accounted for only 20% of the volume of a vesicle lumen having an average diameter of ∼36 nm (see above) and all three aligned to cover over 95% of their respective volumes. Moreover, 70–80% of the volume of each of the 12 assemblies fit within the volume of each model. The finding that most of the volume of each of the 12 assemblies fit into models that occupied 20% of the volume of a vesicle’s lumen, which was only twice the fraction of the luminal volume occupied by an individual assembly, provides strong support for the conclusion that the layout of the luminal assemblies is similar from vesicle to vesicle. The 20–30% of the assembly volumes not fitting in the alignment models were mostly near the vesicle membrane (Figure 4E), indicating a somewhat greater variability in the distribution of the nubs and in the region of the arms attached to them, than in the remainder of the assembly.

### Orientation

**Figure 5:**
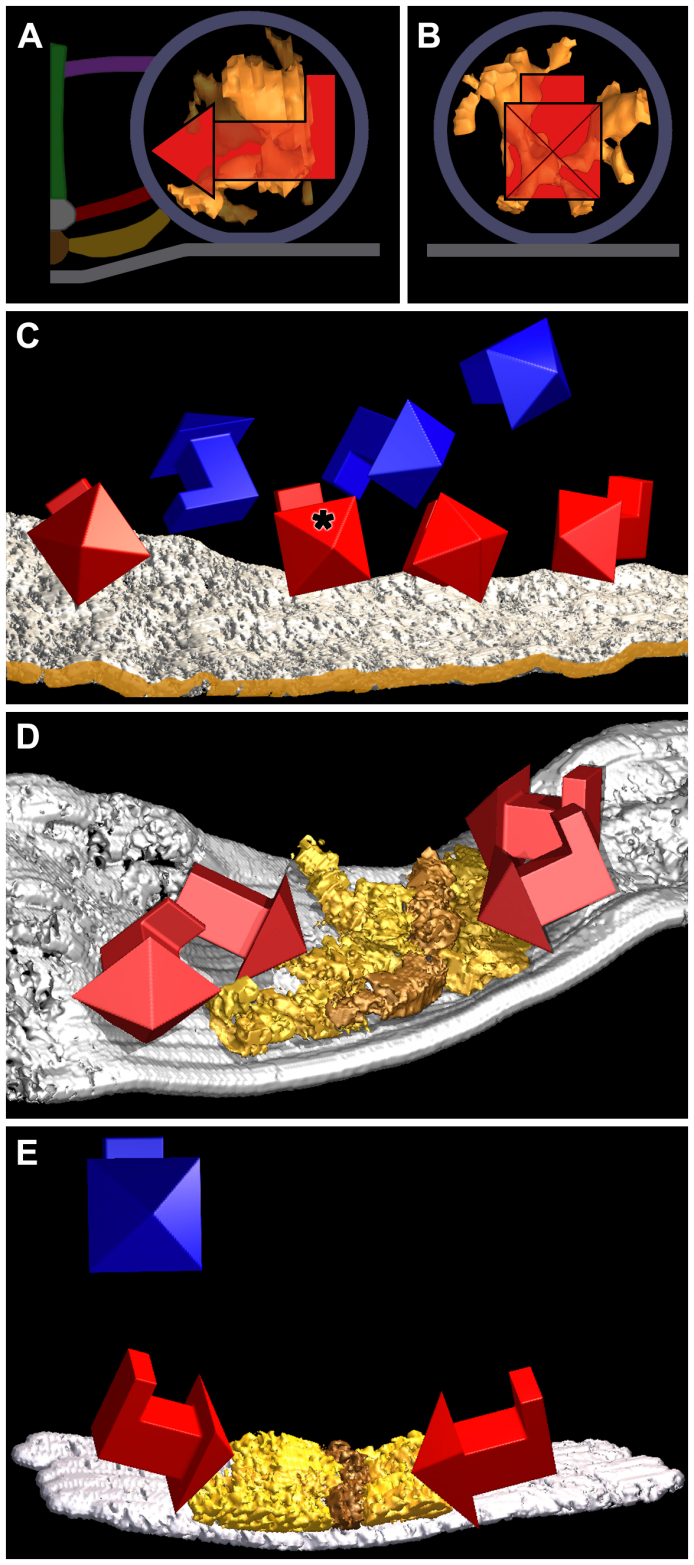
Orientation of the shape of the luminal assemblies. In (A), the surface model of the luminal assembly of the principal vesicle is viewed in the transverse plane of its active zone, while in (B) it is viewed from the median plane of the active zone. The 3D arrow superimposed on the surface model in both (A) and (B) is oriented so its head points to the median plane of the AZM, its shaft is orthogonal to the median plane and parallel to the presynaptic membrane and its tail is vertical to the presynaptic membrane. The vesicle membrane, presynaptic membrane, and AZM macromolecules are schematized for reference. (C) A surface model of the presynaptic membrane in a primary reconstruction, shown in the virtual slices in Figure 3A–E that include the principal vesicle, three other docked vesicles and three undocked vesicles. The edge of the membrane nearest the median plane of the AZM is brown-gold. The 3D arrows show the orientation of the shape of the luminal assembly in the docked (red) and undocked (blue) vesicles relative to that of the principal docked vesicle (asterisk) with respect to the median plane of the active zone and to the presynaptic membrane as shown in (A) and (B). The shape of the assembly in all of the docked vesicles has the same orientation (±30 degrees) as that of the principal vesicle, while the shape of the assembly in undocked vesicles does not share a common orientation. (D) Surface models of the presynaptic membrane and superficial layer of the AZM from the other primary reconstruction. The superficial layer of the AZM, which contains ribs (gold) and beams (brown-gold), is viewed from near the active zone’s transverse plane. There is a slight angular change midway along the AZM’s long axis [11]. The 3D arrows show the orientation of the shape of the luminal assembly in four docked vesicles relative to that of the primary docked vesicle in (A–C) with respect to the median plane of the active zone and presynaptic membrane. The shape of the assembly for three of the docked vesicles had the same orientation (±30 degrees) with respect to the median plane of the active zone and to the presynaptic membrane as the docked vesicles in (C). While the shape of the assembly for the fourth vesicle (arrowhead and shaft lacking a tail) was similarly oriented with respect to the median plane of the active zone, there was not sufficient information to determine its orientation with respect to the presynaptic membrane. (E) Surface models of the presynaptic membrane and superficial layer of the AZM from another reconstruction. The ribs and beams of the AZM are viewed from near the active zone’s transverse plane. The 3D arrows show the orientation of the shape of the luminal assembly in two docked vesicles (red) and one undocked vesicle (blue) relative to that of the primary docked vesicle in (A–C) with respect to the median plane of its active zone and presynaptic membrane. The shape of the assembly for the two docked vesicles had the same orientation (±30 degrees) with respect to the median plane of the active zone and to the presynaptic membrane as the docked vesicles in (C) and (D), while the shape of the assembly in the undocked vesicle did not.

As we were comparing the similarity in the shape of the assembly from vesicle to vesicle, we noted that for docked vesicles, the orientation of the shape with respect to the main body of AZM and the presynaptic membrane was also similar from vesicle to vesicle, while for undocked vesicles it was not. To document such similarities and differences in assembly orientation for the 12 vesicles in the primary reconstructions, we took the individual assembly rotation alignments used to form the gray-scale alignment model of configuration similarities described above, and we calculated how the assembly in each vesicle was oriented with regard to the assembly in every other vesicle. A surface model of the vesicles and presynaptic membrane in each reconstruction was generated, and the luminal assembly for the principal vesicle (shown in Figures 3,4Aa) was chosen as a reference. A simple geometric model of a 3D arrow was made with a head, a shaft and a tail orthogonal to the shaft. The arrow was inserted in place of the principal vesicle in the surface model with the point of the head aimed at the median plane of the active zone, the shaft lying parallel to the presynaptic membrane and orthogonal to the median plane of the active zone, and the tail rising vertical to the presynaptic membrane (Figure 5A,B). Copies of the arrow were individually inserted in place of the other vesicles in the primary reconstructions according to where the coordinates for the arrow in the orientation calculation for the principal vesicle fit into the orientation calculations for their assemblies. As shown in Figure 5C,D for the two active zones in the primary reconstructions, the arrows for the docked vesicles had similar orientations (< ±30 degrees) with respect to the median plane of the active zone and the presynaptic membrane, while the arrows for undocked vesicles (Figure 5C) had no common orientation. The orientation of the luminal assemblies’ shape in two docked and one undocked vesicles in a reconstruction from a muscle other than that used for the primary reconstructions was determined by overlaying the 3D alignment model from the primary reconstructions on the surface model of each luminal assembly, and rotating it to maximize the degree of overlap; the orientation of the alignment model having the greatest degree of overlap with the shape of the assembly provided the orientation of the assembly, and arrows were inserted as described above. As shown in Figure 5E the orientation of the shape of luminal assemblies in the docked vesicles with respect to the median plane of the active zone and to the presynaptic membrane was the same as for the docked vesicles in the primary reconstructions, while the undocked vesicle had no such orientation. A comparable study in other reconstructions showed that the luminal assemblies in only 4 of 33 undocked vesicles were oriented similar to those of docked vesicles in Figures 5C–E.

### The Pairing of Connection Sites of Nubs with those of AZM and non-AZM Macromolecules

**Figure 6:**
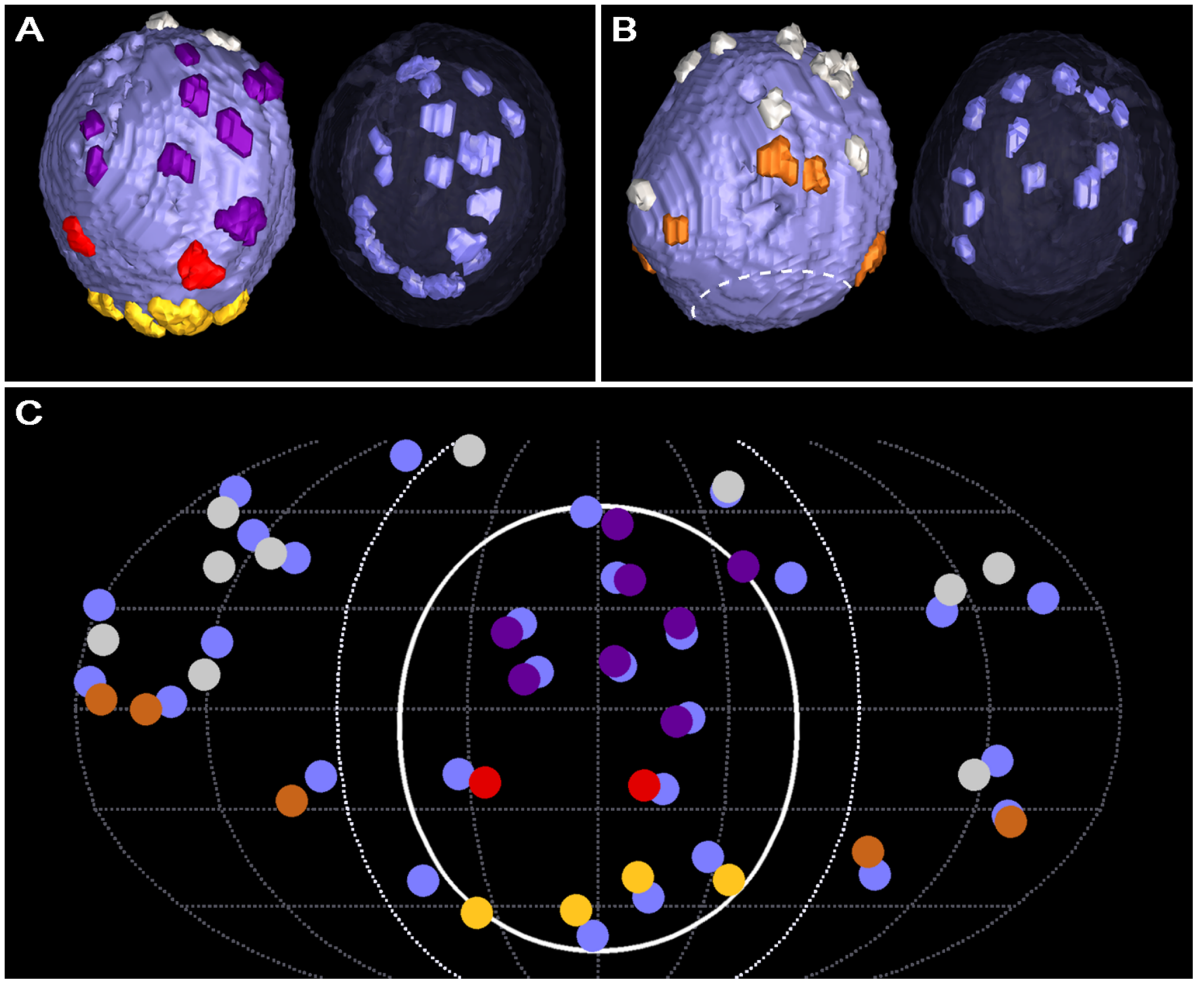
Pairing of connection sites of nubs with connection sites of AZM and non-AZM macromolecules. (A,B) Surface model of the membrane of a docked vesicle in one of the primary reconstructions; (A) shows the hemisphere facing the main body of the AZM and (B) shows the hemisphere facing away from the main body. (A-left, B-left) Three-voxel-thick terminal portions of the AZM’s ribs (gold), spars (red), booms (purple) and pins (copper) and of non-AZM macromolecules (pewter), mark the connection site of each on the outer cytosolic surface of the vesicle membrane (pale blue). The vesicle’s fusion domain is indicated by the dashed line (B). (A-right, B-right) The AZM and non-AZM macromolecules are disabled on the surface model and the membrane has been made almost transparent. Three-voxel-thick terminal portions of the luminal assembly’s nubs (blue) mark the connection site of each on the luminal surface of the vesicle membrane. The distribution of the connection sites of the nubs is non-uniform and appears similar to the asymmetric distribution of the connection sites of the AZM and non-AZM macromolecules, although some of the similarity is obscured in these 2D images by the vesicle’s 3D curvature and the difference in the vesicle’s luminal and outer diameters. (C) Using *x,y,z* coordinates of the connection sites in (A,B), after correcting for the difference between luminal and outer diameters of the vesicle membrane, the relative positions of the centroids of the connection sites of the AZM and non-AZM macromolecules and of the nubs were plotted on a 2D Robinson map. The centroids were overlaid by filled circles slightly smaller in diameter than the connection sites and color-coded as in (A,B). The hemisphere of the vesicle facing the main body of the AZM lies between the bold longitudinal lines. The main AZM binding domain, which covers about 20% of the outer surface area of this vesicle and includes about half of the total nub and AZM/non-AZM macromolecule connection sites on the membrane, is encircled by the solid white line. Each nub is paired with an AZM or non-AZM macromolecule lying opposite to, or slightly offset from, it.

**Figure 7:**
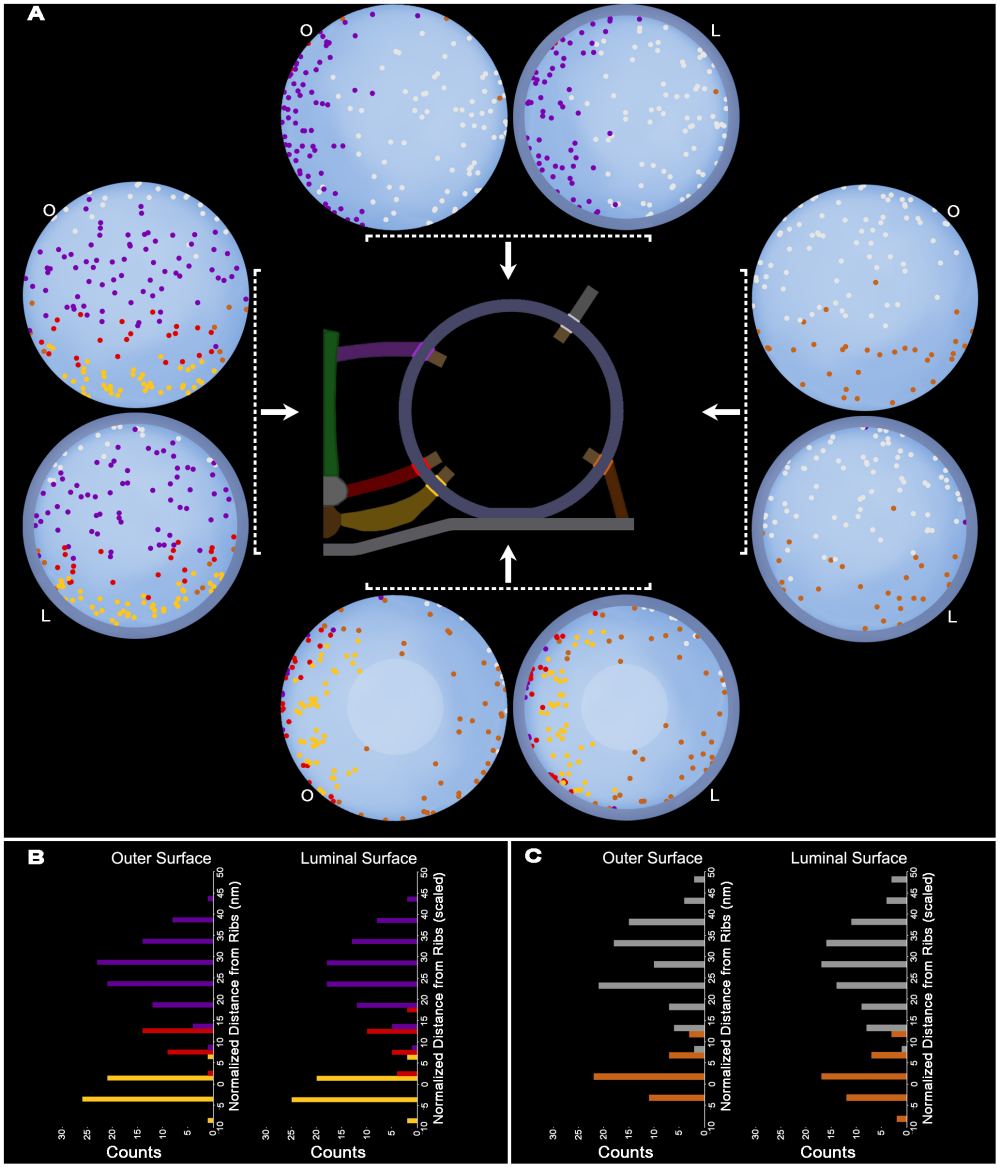
Composite maps of nub, AZM and non-AZM macromolecule connection sites. (A) The centroids of the connection sites of different classes of AZM macromolecules, non-AZM macromolecules and nubs on the membrane of 11 docked vesicles from 4 active zones are plotted on the outer (O) and luminal (L) surfaces of an idealized vesicle. The hemispheres are viewed from (→) or toward (←) the midline of the main body of the AZM or from (↑) or toward (↓) the presynaptic membrane as indicated in the schematic of the active zone. The connection sites of ribs (gold), spars (red), booms (purple), pins (copper) and non-AZM macromolecules (pewter) were plotted on the idealized vesicle, while maintaining their relative positions per vesicle, using a cross correlation method that maximizes the degree of overlap of the rib connections to the outer surface (see Methods). The connection sites of nubs were plotted according to the same method using the nub connection sites paired with rib connection sites as the reference. Nub connection sites are color-coded according to the class of AZM/non-AZM macromolecules with which they were paired. There is an asymmetric distribution of AZM/non-AZM connection sites between the hemisphere facing the median plane of the AZM and the hemisphere facing away from the AZM, which is mirrored by an asymmetric distribution of nubs. Similarly, the asymmetric distribution of AZM/non-AZM connection sites between the hemisphere facing the presynaptic membrane and away from the presynaptic membrane, also is mirrored by an asymmetric distribution of nubs. The distribution of nub and AZM/non-AZM connection sites on the hemisphere facing the presynaptic membrane is greatly affected by the absence of such connections in the fusion domain (within the pale circular area). (B,C) The frequency of the connection sites of the different classes of main body AZM macromolecules on the outer surface and their paired nubs on the luminal surface (B), and non-AZM macromolecules and pins on the outer surface and their paired nubs on the luminal surface (C), as a function of distance from the average rib position per vesicle (see methods). The plots of the nub connections in (B) and (C) are normalized (scaled) for differences between the luminal and outer diameters of the vesicle membrane. The asymmetric distribution of the connection sites of the different classes of AZM and non-AZM macromolecules on the outer surface of the vesicle membrane reflects the asymmetric distribution of connection sites of their paired nubs on the luminal surface of the membrane.

We have reported previously that the fusion domain of the membrane of docked vesicles at frog NMJ’s occupies, on average, about 5% of the total area of the membrane’s outer surface [10]. As a step toward learning whether the distribution of nub connection sites was correlated with the connections sites of the AZM macromolecules, we undertook to measure the fraction of the area of the vesicle membrane’s outer surface occupied by the slightly overlapping sets of the connection sites of ribs, spars and booms. For 11 docked vesicles, taken from muscles fixed with aldehyde or rapid freezing and stained with osmium tetroxide and uranyl acetate in acetone by freeze-substitution, the average outer surface area of their membrane was ∼8300 nm^2^ (8298±1113 nm^2^). These vesicles had, on average ∼4 (4.2±0.4) rib connection sites, ∼2 (2.0±0.4) spar connection sites, and ∼7 (7.0±0.9) boom connection sites. Together, the sets of connection sites on each of the 11 vesicles fell within a circular area that was ∼14% (13.7±3.6%) of the total outer surface area of the vesicle membrane (Figure 6, 7A). Because all connection sites of AZM macromolecules, except for those of pins, were within this area, we refer to it as the vesicles’ main AZM binding domain. The connection sites of the ∼4 (3.9±1.3) pins and ∼9 (8.5±1.8) non-AZM macromolecules on these vesicles were distributed, vesicle to vesicle, over most of the remaining ∼80% of the vesicles surface area, with the connection sites of pins, together with those of ribs, forming a ring around the fusion domain and, therefore, nearer the presynaptic membrane than those of the non-AZM macromolecules (Figure 6, 7A; see also [11]). Thus, the distribution of macromolecule connection sites on the outer surface of the membrane of docked vesicles, although similar from vesicle to vesicle, was highly asymmetric: there were no such connections in the fusion domain, and there was an ∼6-fold greater frequency of connection sites in the main AZM binding domain than in the portion of the membrane beyond the fusion domain and the main AZM binding domain.

For the same 11 docked vesicles, the number of nub connection sites on the luminal surface of the vesicle membrane (23.8±4.4 SD) was similar to the total number of connection sites of AZM and non-AZM macromolecules on the outer surface of the membrane (25.4±3.9 SD), as determined by t-test (p>0.35). In nearly every case (91%) the connection site of a nub was paired with (i.e. opposite or slightly offset from) the connection site of an AZM or non-AZM macromolecule (Figure 6). The small fraction of the cases in which nubs were not paired with AZM/non-AZM macromolecules could well have been due to the failure of the macromolecules to stain. Paired nub connection sites on the luminal surface of the vesicle membrane with the macromolecule connection sites on the outer surface resulted in an asymmetric arrangement for them as it did for the outer connection sites (Figure 6, 7). Accordingly, we found no nub connection sites in the fusion domain, and there was an ∼6-fold greater frequency of nub connection sites in the main AZM binding domain than over the remainder of the luminal membrane surface. Moreover, when we designated nub connection sites on the luminal surface of the membrane of the 11 vesicles according to the class of AZM macromolecules or non-AZM macromolecules on the outer surface with which they were paired, each class of nub connection sites was spatially separate from the next by a distance comparable to that separating the different classes of AZM macromolecules and the non-AZM macromolecules, after accounting for the difference between the luminal and outer vesicle diameters (Figure 6C, 7B,C). Altogether, these findings lead to the conclusion that from docked vesicle to docked vesicle the distribution of nub connection sites on the luminal surface of the vesicle membrane mirrors the connection sites of AZM and non-AZM macromolecules on the outer surface.

### Bands of Stain Spanning the Vesicle Membrane

**Figure 8:**
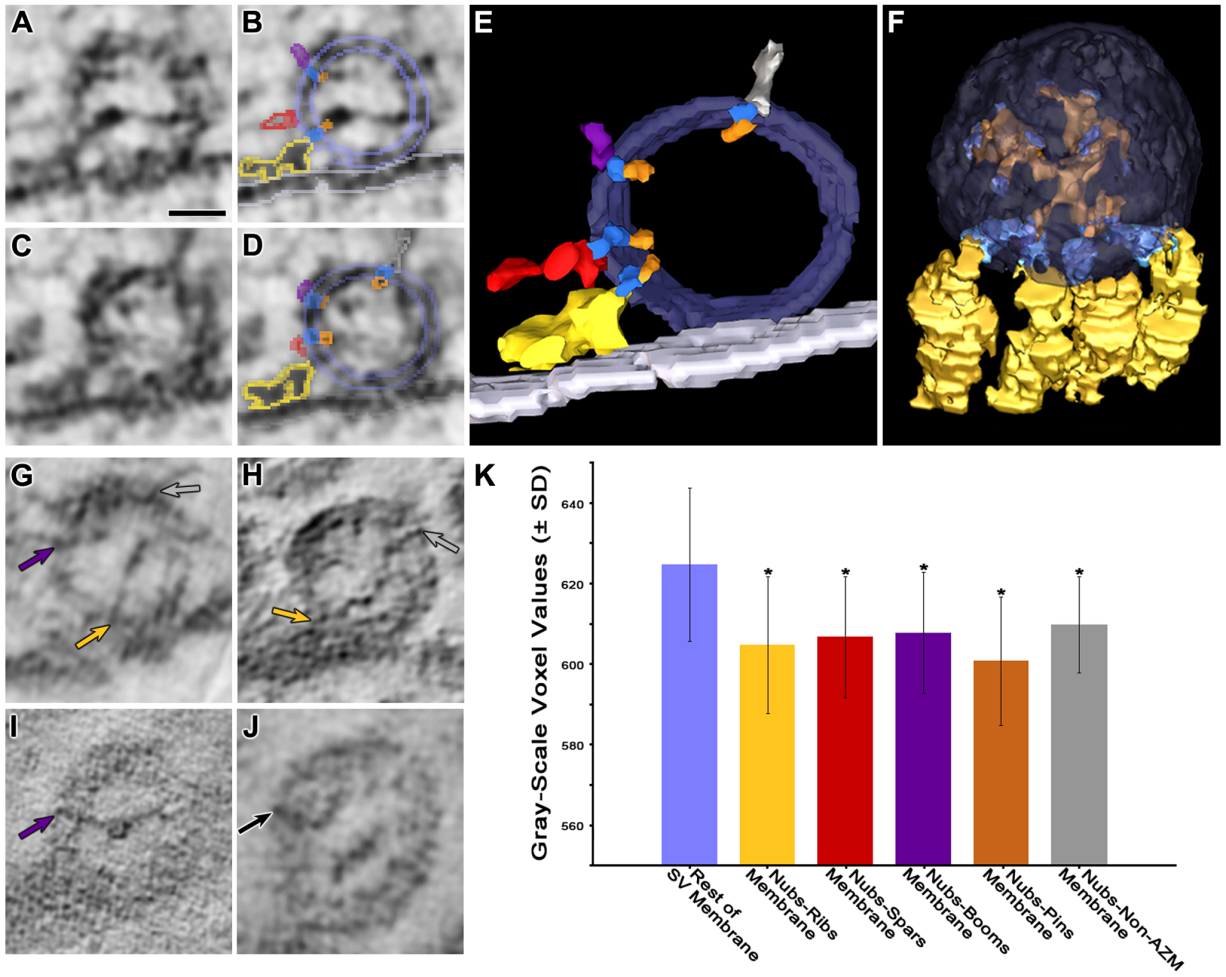
Transmembrane bands of stain linking the connection sites of nubs to AZM and non-AZM macromolecules. (A) Virtual slice ∼2–3 nm thick through a docked vesicle. (B) Same slice as in (A) with color-coded outlines of portions of nubs (orange), transmembrane bands of stain (darker blue), and portions of a rib (gold), a spar (red), and a boom (purple). The pale blue outlines marking the luminal and outer surfaces of the vesicle membrane are an overlay of this section’s contribution to a 3D surface model of the entire vesicle. The nubs are linked to the rib and boom by the transmembrane bands of stain. The band of stain and nub to which the spar is linked is evident in the next virtual slice, shown in (C). (C) A virtual slice adjacent to the one shown in (A) and (B). (D) Same slice as in (C) with outlines of portions of nubs, transmembrane bands of stain and portions of AZM macromolecules and the surfaces of the vesicle membrane color-coded as in (B). Portion of a non-AZM macromolecule is outlined in pewter. Nubs are connected by the transmembrane bands of stain to the spar, boom and non-AZM macromolecule. (E) Surface model ∼10 nm thick generated from a series of virtual slices showing in 3D the nubs linked by transmembrane bands of stain to the rib, spar, boom and non-AZM macromolecule outlined in (B) and (D). The membrane has been made partially transparent to enable viewing the extent of the transmembrane bands in the z-axis. (F) The membrane of the principal vesicle has been made transparent to reveal the luminal assembly of macromolecules (orange). Four ribs (gold) are connected by transmembrane bands of stain (dark blue) to nubs of the luminal assembly at their sites of connection to the luminal surface of the vesicle membrane. The connection sites of other nubs on the membrane were marked by coloring the portion of the nubs within three voxels of their connection site dark blue. (G–I) Virtual slices from different docked vesicles showing nubs linked to transmembrane bands of stain. In adjacent slices (not shown) the transmembrane bands of stain were linked to an AZM or non-AZM macromolecule as indicated by the colored arrows (ribs, gold; booms, purple; non-AZM macromolecules, pewter). (J) Virtual slice from an undocked vesicle showing a transmembrane band of stain linked to a nub. In the adjacent section, the transmembrane band of stain was linked to a non-AZM macromolecule as indicated by the black arrow. Scale bar (A–D,G–J) = 25 nm. (K) For a single vesicle, the frequency distribution of the voxel gray-scale values (ranging from 0, black, to 1000, white) of the narrow region of the vesicle membrane between nub connection sites and the connection sites of opposed AZM and non-AZM macromolecules, i.e. the region containing the transmembrane band of stain, was compared with the voxel gray-scale values for the rest of the synaptic vesicle (SV) membrane. The voxel gray-scale values in the regions between 24 nubs and their opposed ribs (4), spars (2), booms (6), pins (4) and non-AZM macromolecules (8) were on average significantly darker than the rest of the vesicle membrane (asterisks) as determined by ANOVA with a Tukey Post Hoc Test (p<0.05). An additional 7 vesicles were similarly tested and the results are displayed in Table 1 (details of results from this vesicle are shown at #8 in Table 1). Altogether, the findings indicate that, on average, the transmembrane regions linking nubs to AZM and non-AZM macromolecules have a greater density of stained material than the remainder of the vesicle membrane.

The particles of heavy metal that stained structures in our samples were irregular in shape and smaller than the width of the membrane of a synaptic vesicle. Generally, there were higher concentrations of particles at the membranes’ luminal and outer surfaces. However, there were also high concentrations of particles arranged in narrow bands that extended across the width of the membranes. Such transmembrane bands have been observed by electron tomography in the postsynaptic membrane of neuron-neuron synapses, where they are thought to represent stained macromolecules that include neurotransmitter receptors [29]. By visual inspection of the virtual slices through both docked and undocked vesicles in our reconstructions, many of the transmembrane bands of stain appeared to have a diameter similar to that of nubs and to be continuous with nubs at their site of connection to the membrane’s luminal surface (Figure 8). Such bands in the membrane of docked vesicles often extended from the connection site of a nub on the luminal surface of the membrane to the connection site of its paired AZM or non-AZM macromolecule on the outer surface (Figure 8A-I). We undertook to determine the frequency of transmembrane bands connecting nubs to AZM/non-AZM macromolecules by segmenting the bands from serial virtual slices along with the connection sites of nubs and AZM/non-AZM macromolecules and rendering them together with the outlines of the membrane as 3D surface models. We found that for 10 docked vesicles transmembrane bands were clearly evident between 97% of the nub-rib connection sites, 94% of the nub-spar connection sites, 88% of the nub-boom connection sites, and 91% of the nub-pin connection sites.

Some transmembrane bands of stain in the 10 docked vesicles were not associated with macromolecules connected to the luminal and outer surfaces of the membrane. To determine whether transmembrane bands were selectively localized to the paired connection sites of macromolecules on the luminal and outer surfaces, we used the serial virtual slices through the vesicles to compare the frequency distribution of voxel gray-scale values of the narrow region of vesicle membrane between the nub and AZM/non-AZM connection sites to the frequency distribution of voxel gray-scale values throughout the rest of the vesicle membrane. The average voxel gray-scale values for the region of membrane between the connection sites of nubs and each of the classes of AZM and non-AZM macromolecules were significantly different (darker) than the average voxel gray-scale value for the remainder of the vesicle membrane (Figure 8K; Table 1), which would be expected if the transmembrane bands of stain were preferentially localized to regions of the vesicle membrane between the connection sites of nubs and the connection sites of AZM and non-AZM macromolecules. Because the staining of these transmembrane bands was similar to and usually appeared continuous with that of the paired macromolecules connected to the luminal and outer surface of the membrane, we conclude that each band of stain marked a membrane macromolecule linking a nub to either an AZM or a non-AZM macromolecule. The small fraction of the cases in which we did not visually detect bands of stain between the connection sites of nubs and AZM/non-AZM macromolecules was probably owing to the capriciousness of the stain. We did not undertake a systematic study to determine whether the transmembrane bands of stain continuous with nubs in undocked vesicles were also linked to non-AZM macromolecules that connect to the outer surface of these vesicles, but we noted in many cases that they were (Figure 8J).

**Table 1:** Voxel gray-scale values for the narrow regions of membrane between the connection sites of nubs and connection sites of their paired AZM/non-AZM macromolecules and for the rest of the membrane of eight synaptic vesicles.

SV	Rest of Membrane		Nubs-Ribs		Nubs-Spars		Nubs-Booms		Nubs-Pins		Nubs-non-AZM	
	N_v_	Avg±SD	N_v_	Avg±SD	N_v_	Avg±SD	N_v_	Avg±SD	N_v_	Avg±SD	N_v_	Avg±SD
**1**	72082	581±41	1658	558±40	512	565±40	1432	578±42*	610	561±42	1653	560±40
**2**	76582	500±105	652	483±99	310	466±82	568	445±86	584	415±85	2130	431±90
**3**	70993	481±102	914	444±96	598	424±95	1126	438±89	772	418±93	2264	417±85
**4**	77597	486±97	791	433±85	389	425±82	1259	425±88	804	430±83	1330	420±89
**5**	69063	491±103	649	438±79	559	443±97	931	407±89	547	421±74	995	391±90
**6**	64417	460±86	759	413±77	362	401±70	1150	391±85	555	400±84	1764	397±88
**7**	72812	432±87	911	370±76	220	377±71	786	373±76	259	364±77	663	356±75
**8**	81576	625±19	680	605±17	325	607±15	707	608±15	575	601±16	951	610±12

N_v_, number of voxels; Avg±SD, average gray-scale value (ranging from 0, black, to 1000, white) ± Standard Deviation (SD). Average gray-scale values for the regions between nubs and AZM/non-AZM macromolecules per vesicle are significantly lower (darker) than for the rest of its membrane, except for one value (asterisk), as determined by ANOVA analysis with Tukey Post Hoc test (p<0.05).

## Discussion

**Figure 9:**
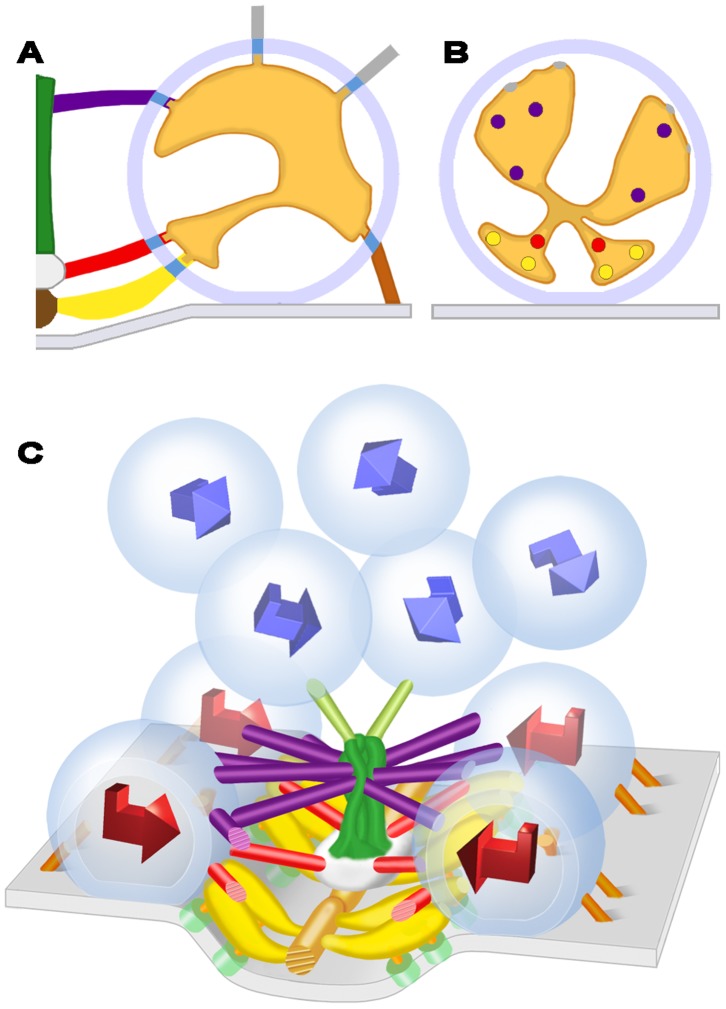
Schematized relationships of the luminal assembly of macromolecules at frog NMJ’s. (A) Profile of a vesicle (pale blue) docked on the presynaptic membrane (gray) viewed in the transverse plane of the active zone. AZM macromolecules, including a rib (gold), a spar (red), a boom (purple) and a pin (copper), connect to the outer surface of the vesicle membrane as do non-AZM macromolecules (pewter). The luminal assembly of macromolecules is orange. Nubs arise from the luminal assembly’s arms to connect to the vesicle membrane, where they are linked to macromolecules that span the membrane (dark blue) and connect to the AZM and non-AZM macromolecules. (B) Profile of a docked vesicle viewed from the active zone’s median plane. The arms of the luminal assembly radiate from a point near the center of the vesicle and are larger near the vesicle membrane than at the vesicle’s center. The arms in the hemisphere facing the presynaptic membrane are shorter than those away from the presynaptic membrane. Colored spots on the luminal assembly mark regions connected by nubs and their membrane spanning macromolecules to specific classes of AZM and non-AZM macromolecules, as in (A). C) 3D arrangement of docked and nearby undocked vesicles relative to the AZM and presynaptic membrane. The orientation of the stereotypic shape of the luminal assembly with respect to the median plane of the AZM and presynaptic membrane is indicated by 3D arrows. The orientation is the same for docked vesicles (red arrows) while for undocked vesicles (blue arrows) it is not. Thus, in order for an undocked vesicle to replace a docked vesicle that fuses with the presynaptic membrane during synaptic transmission, it must, typically, rotate so the appropriate vesicle membrane macromolecules linked to the luminal assembly (A,B) can sequentially interact with the different classes of AZM macromolecules that direct it to the docking site on the presynaptic membrane.

We show by electron tomography that the lumen of synaptic vesicles at appropriately stained frog NMJ’s contains an assembly of macromolecules. The luminal assembly occupies about 10% of the lumen’s volume, and it has a chiral bilateral shape that is similar from vesicle to vesicle. As schematized in Figure 9A,B irregular arms radiate from near the lumen’s center to connect by nubs to the luminal surface of the vesicle membrane. The number of nub connection sites on the membrane is similar vesicle to vesicle, and, at these sites, the nubs are linked to stained macromolecules that span the membrane. For docked vesicles, the orientation of the luminal assembly with respect to the presynaptic membrane and the median plane of the active zone is similar vesicle to vesicle, while in undocked vesicles it is not (Figure 9C). Moreover, for docked vesicles, the nub connection sites on the luminal surface of the vesicle membrane are paired with, and linked by their transmembrane macromolecules to, AZM and non-AZM macromolecules at the connection sites of these macromolecules on the outer surface of the membrane. Accordingly, there is a stereotypical asymmetric distribution of nub connection sites on the luminal surface of the membrane of docked vesicles that mirrors the asymmetric distribution of the connection sites of AZM and non-AZM macromolecules on the outer surface. We have found in other experiments (Xu and McMahan, unpublished) that the distribution of nub connection sites on the membrane of undocked vesicles is similar to that of docked vesicles, which is consistent with our findings here that the shape of the luminal assemblies and the number of nub connection sites in undocked vesicles is similar to that of docked vesicles. Altogether, our findings on the structure and associations of the luminal assembly in synaptic vesicles at frog NMJ’s bear directly on the problems of how different proteins are arranged in the vesicle membrane at this synapse, and how the membrane of undocked vesicles becomes associated with AZM and presynaptic membrane during docking. They are also relevant to the problems of how transmitter is released from the vesicles when their membrane fuses with the presynaptic membrane during synaptic transmission and how the fused vesicle membrane is, then, recycled. 2D electron micrographs from freeze-fracture studies on synapses in different regions of the nervous system and in different animal species [30]–[37], as well as on the NMJ of the frog [38], show that the membrane of synaptic vesicles contains multiple particles, each of which could represent one or more of the transmembrane macromolecules we detected by electron tomography. Other studies, using different methods of analysis, on isolated synaptic vesicles from the electric organ of a marine ray and from the rat’s brain indicate that the lumen of these vesicles contains macromolecules [39], [40]. Such findings, together with evidence that the assortment of synaptic vesicle proteins involved in docking is much the same throughout the nervous system [5], [20], make it likely that our observations and conclusions have general applicability.

Biochemistry indicates that proteins in the lumen of synaptic vesicles are luminal portions of ones that span the vesicle membrane [20]. We propose that the luminal assembly of macromolecules we describe at frog NMJ’s is composed of aggregates of the luminal portions of such membrane spanning proteins, that most, if not all, of these portions enter the lumen at the connection sites of the assembly’s nubs on the luminal surface of the vesicle membrane, and that membrane spanning proteins giving rise to the luminal portions are components of the transmembrane macromolecules linked to the nubs. By extension, the nearly constant chiral shape of the luminal assembly and the similarity in number and distribution of nub connection sites from vesicle to vesicle indicate that those proteins in the transmembrane macromolecules linked to nubs have a particular asymmetric arrangement common to both docked and undocked vesicles. The association of the luminal portions of vesicle membrane proteins in the assemblies very likely helps anchor the proteins in position within the membrane and, in so doing, provides the membrane with tensile strength to accommodate forces imposed on the vesicles during, for example, docking [11].

Several vesicle membrane proteins having a luminal portion are known to also have a cytosolic portion that interacts with other cytoplasmic components. These proteins include synaptobrevin and synaptotagmin, the cytosolic portions of which are involved in vesicle docking and fusion, as well as SV2, and synaptophysin [41]–[44]. Based on their primary structure, the luminal portions of synaptobrevin, synaptotagmin and synaptophysin, could extend into the lumen for only a small fraction of its diameter [45]–[48]. On the other hand, the length of the luminal portion of SV2 is much greater than the diameter of the lumen [44]. Thus, folded luminal portions of SV2 are likely to serve as the luminal assembly’s mainstay for the attachment of the luminal portions of the other proteins. The dissociation characteristics of vesicle proteins, including synaptobrevin, synaptotagmin and SV2, during biochemical isolation indicate they are coherent in their native state [49], which would be expected if they were components of membrane macromolecules and/or a luminal assembly of macromolecules.

Based on spatial requirements for the interaction of the cytosolic portions of the synaptic vesicle’s synaptobrevin and synaptotagmin with the cytosolic portions of the presynaptic membrane’s syntaxin and SNAP-25 during docking, we have suggested previously [11] that, for docked vesicles, these portions are components of the AZM’s ribs and pins. We propose here that the transmembrane macromolecules and nubs linking the ribs and pins to the luminal assembly contain membrane spanning and luminal portions of synaptobrevin and synaptotagmin and, perhaps, other membrane-spanning proteins such as SV2. Similarly, the transmembrane macromolecules and nubs linking the AZM’s spars and booms to the luminal assembly of docked vesicles must also contain membrane spanning and luminal portions of proteins having cytosolic portions involved in docking, while nubs linking non-AZM macromolecules to the luminal assembly contain membrane spanning and luminal portions of proteins having cytosolic portions involved in general aspects of vesicle trafficking. We have proposed elsewhere [11] that vesicle proteins lacking luminal portions and known to be involved in docking, such as Rab3a, or trafficking in general, such as synapsin, contribute to the AZM and non-AZM macromolecules, respectively. Such proteins may also be a part of the transmembrane macromolecules that link the AZM and non-AZM macromolecules to the luminal assembly along with those proteins having luminal portions.

Evidence that the distribution of nubs and their transmembrane macromolecules is the same in undocked vesicles as it is in docked vesicles, where most of the nubs are linked by their transmembrane macromolecules to AZM macromolecules in the vesicles’ AZM binding domain and/or ring their fusion domain, leads to the conclusion that the connection sites of AZM macromolecules and the fusion domain are predetermined on undocked vesicles. Thus, in order for undocked vesicles to sequentially form connections with booms, spars, and ribs and pins [11], which leads to docking on the presynaptic membrane, they need to move in a way that results in the alignment of the appropriate nubs and their transmembrane macromolecules with the AZM macromolecules so their proteins can interact. Because in undocked vesicles the shape of the luminal assembly, which is indicative of the distribution of the nubs and their transmembrane macromolecules, does not have a specific orientation toward the main body of the AZM and the presynaptic membrane, as it does in docked vesicles, such alignment must involve vesicle rotation. One scheme for how the vesicles in the cloud of undocked vesicles adjacent to the active zone may move to achieve the alignment is as follows. After the membrane of docked vesicles fuses with the presynaptic membrane to mediate synaptic transmission, the cytosolic portions of its proteins connected to the AZM and non-AZM macromolecules dissociate from them [5], [11]. The vesicle membrane, then, flattens into the presynaptic membrane and moves laterally beyond the active zone to be retrieved for recycling [38], [50]. The calcium that enters the terminal to trigger the fusion of the docked vesicles with the presynaptic membrane also brings about the disruption of the macromolecules that link undocked vesicles to each other in the cloud, so the vesicles are free to move [24], [25], [51]. The initial movement may be random Brownian motion**,** which would result in vesicle rotation and favor the vesicles’ displacement from the high vesicle density of the cloud to the relatively low vesicle density of the active zone due to the fusion of the membrane of docked vesicles with the presynaptic membrane and its lateral displacement. Once an undocked vesicle is sufficiently close to a vacated set of booms, and it has rotated so the appropriate set of nubs and their transmembrane macromolecules are aligned with the booms, the cytosolic portions of the proteins in the transmembrane macromolecules can then, according to their affinity, interact with proteins in the booms to constrain Brownian movement. The stability of the interactions and the strength of constraint could increase as the number of booms involved in such interactions increases. Moreover, force generated by these interactions might help bring the adjacent set of nubs and their transmembrane macromolecules close enough to the vacated spars for their proteins to interact. This sequence could be repeated so that, ultimately, the cytosolic portions of synaptobrevin and synaptotagmin in the set of nub-linked transmembrane macromolecules containing them interact with the cytosolic portions of syntaxin and SNAP-25 in the vacated ribs and pins. The force generated by these interactions would not only bring the fusion domain of the vesicle’s membrane into precise alignment with the presynaptic membrane, but it also would lead to the two membrane’s making direct contact. The localization of docking proteins to macromolecules having an arrangement in the vesicle membrane that mirrors the arrangement of AZM macromolecules would minimize the number of protein copies required for the docking process to occur. To our knowledge there is no biochemical evidence to indicate there is a region of the synaptic vesicle membrane specialized for forming a fusion pore with the presynaptic membrane during synaptic transmission. However, this possibility is raised by our structural evidence that the portion of the membrane that will form the pore is predetermined in undocked vesicles and that one of the roles of the AZM during docking is to bring this portion into direct contact with the presynaptic membrane. The specialization could be in the nature of the membrane proteins or lipids or both. The lipid composition of vesicle membranes is known to influence the probability of their calcium-mediated fusion with plasma membranes in certain non-neural cells [52], [53].

Biochemical and electrophysiological studies on cholinergic synaptic vesicles, including those at the frog’s NMJ, indicate that the luminal proteins, particularly SV2, have abundant carbohydrate residues, and that the carbohydrate residues adsorb the neurotransmitter acetylcholine [39], [54]. The rate of dissociation of acetylcholine from this glycomatrix after vesicle fusion with the presynaptic membrane is thought to significantly influence the timing of its exocytosis during synaptic transmission. Our methods of tissue staining did not permit us to detect extended carbohydrate residues, but the luminal assembly of macromolecules is likely to serve as the protein backbone of the glycomatrix. Thus, the stereotypic shape of the luminal assembly indicates that the carbohydrate residues have the same compartmentalized distribution within the lumen from vesicle to vesicle. Such compartmentalization would be of particular interest if acetylcholine molecules and molecules of ATP, a cotransmitter in cholinergic synaptic vesicles [55]–[58], were localized to different compartments of the glycomatrix according to, for example, specific sites of loading for each. The stereotypic orientation of the luminal assembly in docked vesicles might enable the timing of dissociation from the different compartments, after the vesicles fuse with the presynaptic membrane, to be the same from one vesicle to the next.

The fusion of vesicles with the presynaptic membrane and the recycling of their membrane for fusion again can occur in less than 1 minute at frog NMJ’s [59], and at other synapses [60], [61]. Given the orderly arrangement of the components of the luminal assembly and their associated membrane macromolecules in docked and undocked vesicles and the predicted involvement of this arrangement in vesicle docking, it is reasonable to suggest that such polarity is fully established in undocked vesicles shortly after they are reformed. It may be that when docked vesicles fuse with the presynaptic membrane, the shape of the luminal assembly is temporarily altered to accommodate the flattening of the vesicle membrane into the presynaptic membrane while its different macromolecular components remain associated to help maintain the relative positioning and constant copy number [62] of the membrane macromolecules to which they are linked. The imaging methods we have used here should reveal at NMJs fixed during synaptic activity (e.g. [11]) whether the macromolecules of the luminal assembly remain associated with the membrane of fused vesicles as they flatten into the presynaptic membrane and whether they are present as the vesicles reform [63], [64].

## Materials and Methods

### Ethics Statement

The animal experimentation described here was approved by Stanford University’s (Protocol Number 10505) and Texas A&M University’s (AUP Number 2011–18 ) administrative panels on laboratory animal care (IACUC), which oversees the use of animals according to U.S. federal regulations.

### Tissue Preparation

We used the paired cutaneous pectoris muscles in *Rana pipiens*, which are situated just beneath the skin of the frog’s chest. Muscles from 10 frogs, about 5 cm nose-rump length, provided the data described here. Each frog was deeply anaesthetized in tricaine methanesulfonate (MS-222, Sigma Chemical, St Louis, Missouri) and pithed in both directions prior to tissue removal. The cutaneous pectoris muscles are broad and flat, and 1–3 muscle fibers thick, which favors rapid and uniform fixation and staining.

#### Muscles fixed and stained with glutaraldehyde, osmium tetroxide and uranyl acetate at room temperature [8], [11]

Muscles were immediately exposed in terminally anesthetized (MS-222, Sigma Chemical, St Louis, Missouri) and pithed frogs. Under a dissecting microscope, 1% glutaraldehyde (Ted Pella, Inc., Redding, California) in Millonig’s phosphate buffer (230 mOsM total, pH 7.2) was injected beneath the muscles and dripped onto their superficial surface several times over 15 min. The muscles were removed from the frog, pinned flat in a Sylgard 184 (Dow Corning, Midland, Michigan) coated petri dish containing 1% glutaraldehyde in phosphate buffer (230 mOsM, pH****7.2) and placed on a shaker for 50 min, washed with phosphate buffer for 15 min, tris buffer (230 mOsM, pH 7.2) for 15 min, and phosphate buffer for 20 min. The muscles were then further fixed and stained for 1 hr in 1% osmium tetroxide in phosphate buffer (230 mOsM total; pH 7.2), washed for 1 hr in H_2_O, stained 1 hr in saturated aqueous uranyl acetate, dehydrated in increasing concentrations of ethanol and embedded flat in a wafer of Eponate 12 (Ted Pella, Inc., Redding, California) less than 1 mm thick.

#### Muscles fixed and stained with osmium tetroxide and uranyl acetate at room temperature

Muscles were removed from anesthetized and pithed frogs and pinned out in a petri dish containing Ringer’s solution. They were then fixed and stained for 1 hr in 1% osmium tetroxide in phosphate buffer (230 mOsM total; pH 7.2) before washing in H_2_O, staining in saturated aqueous uranyl acetate and further processing as above.

#### Muscles fixed and stained by rapid freezing and freeze-substitution with osmium tetroxide and uranyl acetate

1) Twelve years ago, when we began this study, Thomas Reese and John Heuser gave us an Araldite embedded block of *Rana pipiens* cutaneous pectoris muscle that had been prepared by rapid freezing for one of their studies published 20 years previously [38]. The freezing was accomplished by mounting the muscle on a freezing press and plunging it onto an ultrapure copper block pre-cooled to 4°K. Freeze-substitution with 4–5% osmium tetroxide in anhydrous acetone occurred over a 4–6 hour warm-up to room temperature. The muscle was then further stained with 1% uranyl acetate in acetone for 2–4 hours, before it was embedded in Araldite for sectioning. This block of muscle contained 12 NMJ’s devoid of obvious ice crystal damage. Two of the active zones were used to generate the principal reconstructions described here. 2) All other muscles initially fixed by rapid freezing were prepared as follows. They were pinned out in Sylgard coated petri dishes and bathed in Ringer’s solution containing 10 mg/ml tetrodotoxin (Sigma-Aldrich Co., St. Louis, Missouri), which helped reduce muscle contraction during mounting. Regions of innervation were identified in the muscles with the dissection microscope. 2–3 mm^2^ portions containing them were cut out and placed between copper mounting carriers (600 µm deep). The carriers were then transferred to a Bal-Tec Ltd. HPM 010 (Lichtenstein) freezing apparatus. Freezing was done at liquid nitrogen temperature and under high pressure. Freeze-substitution of 2% osmium tetroxide together with 0.1% anhydrous uranyl acetate in acetone was done according to the method in [65], and the muscles were embedded in Eponate 12 for sectioning.

#### Muscles fixed with glutaraldehyde at room temperature and further fixed and stained by rapid freezing and freeze-substitution with osmium tetroxide and uranyl acetate

The muscles were fixed with glutaraldehyde in situ and pinned out flat in a Petri dish containing phosphate buffer as described above. 2–3 mm^2^ portions containing regions of innervation were cut out and placed between copper mounting carriers. Rapid freezing in the Bal-Tec freezing apparatus, freeze-substitution of 2% osmium tetroxide and 0.1% uranyl acetate and embedding proceeded as above.

### Sections

Regions of the muscles containing NMJ’s were identified in the wafers of Eponate 12 at x400 magnification with a dissecting microscope, and blocks were cut out and mounted for sectioning. The sections varied from ∼50 nm to 120 nm in thickness based on measurements from the reconstructed volumes. They were stained for 10 min with saturated uranyl acetate in methanol, rinsed with water, stained again with Reynolds lead citrate for 10 min, and again rinsed with water. Staining the tissue sections with uranyl acetate and lead citrate significantly enhanced the contrast of structures already stained by osmium tetroxide and uranyl acetate before embedding, when viewed in the electron microscope.

### Data Collection

Datasets were collected at a magnification ranging from 29,000× to 125,000× using one of two electron microscopes designed for automatic data acquisition: 1) a Philips Tecnai T20 electron microscope (FEI Company Hillsboro, Oregon) equipped with a 1024×1024 CCD (Gatan, Inc., Pleasanton, California) in the laboratory of Dr. David Agard at the University of California, San Francisco; and 2) an FEI TF30 Polara electron microscope (FEI Company Hillsboro, Oregon) equipped with a 2048×2048 Tietz TemCam-F224HD CCD (Tietz Video and Imaging Processing Systems GmbH, Gauting, Germany) in our own laboratory at Stanford University. The stage on each microscope was cooled to liquid nitrogen temperature to reduce specimen shrinkage. Datasets consisted of images taken at 1-degree tilt intervals to ±60 or ±70 degrees along a single tilt axis. The active zones used for data collection were selected so that each of the three different cardinal active zone planes were represented in our sample. This enabled us to account, during data analysis, for any missing wedge artifact resulting from the method of data collection.

### Reconstructions

The tilt images were aligned automatically using 5 or 10 nm gold colloid (British Biocell International, Cardiff, U.K.) deposited on one or both sides of the sections as fiducial markers before data collection. For the datasets used in the current study, the scheme provided an average accuracy of 1.03±0.38 pixels RMS (0.76±0.55 nm RMS). The reconstructions were made by a weighted back-projection method. Both the alignment and reconstruction algorithms are in the unified software package for electron tomography, EM3D (em3d.stanford.edu) [9], [66]. The 3D spatial resolution was 2–3 nm for high contrast structures such as the cytoplasmic and extracellular layers of the plasma membrane [66].

### Virtual Slices, Segmentation and Surface Models

Virtual slices through the reconstructed tissue sections were 1 voxel thick. Depending on the dataset, the virtual slice thickness represented 0.52 nm to 1.2 nm of a tissue section’s thickness. The images of virtual slices in Figures 2 and 8 were formed by the summation of multiple virtual slices to the thicknesses that are specified in the figure legends. When necessary, the angular orientation of the slice plane was adjusted to maximize contrast boundary discrimination of the structures under study.

Structures were segmented from the reconstructions by using a combination of manual and semi-automatic methods in EM3D to define individual volumes-of-interest (VOIs; [9]). For the presynaptic membrane and synaptic vesicles, which were heavily stained and had a simple geometry, a semi-automatic scheme was used and manually adjusted as necessary. For structures that had a complex geometry and light to moderate stain, VOIs were defined by manually marking a closed path on the series of slices in which they were included. The VOIs were slightly larger than the structures that they enclosed to allow accurate and complete isodensity-surface calculations for the surface models.

We used EM3D to render a surface model from each VOI. The rendering was done using a gray-scale value that minimized the mean spatial uncertainty averaged across the whole area of the model. Surface models generated in this way had a spatial resolution equal to the resolution of the reconstructed volumes [9].

### Alignment Models

#### General

The gray-scale density and the two surface model approaches relied on a reference-free alignment algorithm [28]. In the first stage of alignment, a ‘random approximation’ was derived by the alignment and averaging of successive luminal assemblies; one-by-one vesicle luminal assemblies were aligned and then averaged into the model, creating a new partial model at each step. When all of the luminal assemblies had been averaged to form the initial complete model, it was refined by individually removing each assembly from the model, re-aligning, re-introducing the removed assembly, and then re-averaging. The refinement step was repeated five times, at which point the luminal assemblies aligned to the same positions resulting in the final alignment models described in Results. The gray-scale density alignment technique is reliable, but tends to align areas of high density at the potential expense of an overall large area of overlap. The surface model alignment technique ensures that large areas of overlap were not obscured by regions of high density, but may over-represent the alignment of fine structures and noise at the expense of areas of high density.

#### Gray-scale density alignment

Registration of the luminal assemblies was based on the alignment of the gray-scale densities within their VOIs. Likely rotation and translation peaks for alignment models and luminal assembly VOIs were first found visually using surface models. Real space overlapping of the models with the luminal assembly VOIs was done by rotating the individual luminal assembly VOIs ±30 degrees (1 degree intervals) and moving the rotated luminal assembly VOIs translationally ±3 voxels (1 voxel intervals). Scoring, leading to the final alignment model (red model in Figure 4D), was based on the product of the overlapping volumes at each rotation and translation.

#### Surface models alignment

Registration of the luminal assemblies based on the alignment of their surface models was carried out using the ICP (Iterative Closest Point) algorithm [67]. Surface models generated in the IDL version of EM3D were exported to the ‘.ply’ data format. Individual luminal assemblies were then aligned to form the models starting at 20 degree step points in the *x,y,z* rotation using the software package scanalyze (http://graphics.stanford.edu/software/scanalyze/). Best-matches were determined by the minimization of the distance between the alignment models and surface models. The reference-free alignment and refinement approach (above) was used to build two final alignment models. One final model was based on registering luminal assemblies in the same order as in the final gray-scale density alignment model (blue model in Figure 4D); the other model was based on registering luminal assemblies in a random order to rule out founder bias (green model in Figure 4D).

### Measurements

#### Vesicle diameters

The outer and luminal diameters of synaptic vesicles were determined by a method we used elsewhere [10]. Serial virtual slices made through reconstructed volumes in their *x-y* plane were used to identify the slice for each vesicle that passed through its equator. Because the vesicles were not perfect spheres, diameters to the outer surface, or luminal surface, of the vesicle membrane were measured for each vesicle along four separate axes (∼45° increments) in the *x-y* plane and one axis in the *z*-axis, and expressed as an average.

#### Vesicle membrane surface areas

The surface area of a synaptic vesicle (SA_SV_) was calculated based on measurements of the diameter of the synaptic vesicle (d_SV_) using the following: 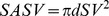
To determine the area of the main AZM binding domain (SA_AZM_) on the vesicle surface, the main AZM binding domain was treated as the surface area of a spherical zone, which is defined as a region of a sphere cut off by a plane. The diameter of the circle at the base of the spherical zone (d_AZM_) for individual vesicles was the maximum distance from the average connections of the ribs to those of the furthest boom. d_AZM_ was used to calculate the SA_AZM_ as follows:
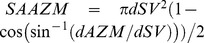
_._

#### Volume overlap of alignment models

Each of the alignment models described above were represented as surface models and aligned to maximize the degree of overlap in 3D space, as described in the ‘ICP Registration of Surface Models’ method above. The number of voxels that overlapped within the shells of the surface models following this alignment was then extracted using IDL software, and used to calculate the percentage of volume overlap between the different alignment models.

#### Distances between connection sites of different classes of macromolecules

The direct distance from the centroid of each connection site to the closest point on the presynaptic membrane was measured using the ‘proximity tool’ in EM3D and then normalized to the average position of the rib connection sites on the outer surface, or the connection sites of the nubs paired with ribs on the luminal surface, per individual vesicle. For the connection sites on the luminal surface, the measurements were normalized to the outer diameter of the vesicles’ membrane by dividing each distance by the ratio of the luminal diameter/outer diameter, so that statistical analyses could be performed between the distributions of connection sites of AZM and non-AZM macromolecules to the outer surface and the nubs paired with the AZM and non-AZM macromolecules on the luminal surface. These normalized measurements were plotted in Figure 7B,C.

### Robinson Projection

To visually compare the relative positions of connection sites on the outer surface of a vesicle membrane to the nub connections on the luminal surface of the membrane, we converted 3D spherical plots to 2D pseudo-cylindrical, or Robinson, projections [68]. The position of the spatial coordinates of the centroids (*x,y,z*) of connection sites of AZM and non-AZM macromolecules on the outer surface of synaptic vesicle membranes, and nubs on the luminal surface of the vesicle membranes were first plotted onto an idealized sphere based on the outer and luminal vesicle diameters using IDL software. By plotting these coordinates on an idealized sphere, differences in the luminal and outer diameters of the vesicle were normalized. The 3D plots of the centroids on the idealized sphere were then warped and expressed on a 2D Robinson projection using the MAP_SET procedure of IDL 7.0.

### Composite Maps of Connection Sites on Docked Vesicles

The connection sites on the outer and luminal surfaces for each synaptic vesicle were mapped independently onto a unit sphere based on the spatial coordinates of the centroids of connection sites and the diameter (outer and luminal) of the individual vesicle to normalize the variability of vesicle diameters according to the methods of [11]. All of the vesicles were then rotated so their connection sites faced the same direction, which provided a rough alignment. For fine-alignment of the vesicles to the position of the rib connections on the outer surface, or the nubs paired with ribs on the luminal surface (in *x,y,z* coordinates), the degree of overlap of rib connections, or nubs paired with ribs, for all vesicles was maximized based on the following equation:



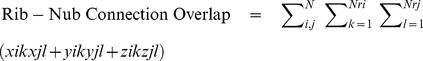
; where *i*≠*j*, *N* is the number of vesicles, *Nri* is the number of Ribs or Nubs paired with Ribs of vesicle *i*, *Nrj* is the number of Ribs or Nubs paired with Ribs of vesicle *j*. To calculate the rotation angle with maximized values for *Rib-Nub Connection Overlap*, we used Euler’s rotation theorem [69]. All vesicles were then rotated to their calculated value, and all AZM connections were plotted onto a common unit sphere shown in Figure 7A.

### Regional Voxel Gray-scale Stain Density of the Vesicle Membrane

We determined the distributions of gray-scale values of voxels included in segmented VOIs. The gray-scale values ranged from 0 to 1000 in arbitrary units, where 0 represented black and 1000 represented white. The region of the membrane between the connection site of each AZM and non-AZM macromolecule and their paired nubs were manually segmented to define its VOI. The entire vesicle membrane was also segmented, and the VOIs from the regions between the connection site of each AZM and non-AZM macromolecule and their paired nubs were subtracted to define the VOI of the rest of the vesicle membrane. The gray-scale value of each voxel that composed the VOIs were extracted using EM3D, and these values for the membrane regions between each class of AZM and non-AZM macromolecule were pooled; to control for the variability of staining between datasets, each vesicle was treated as an individual experiment for statistical purposes. The gray-scale distributions for the membrane regions between the different classes of AZM and non-AZM macromolecules were compared to the rest of the vesicle membrane by ‘Analysis of Variance’ (ANOVA) with the Tukey Post Hoc Test.

### Figure Layouts

Figure layouts were prepared using Adobe Photoshop CS3 (Adobe Systems, San Jose, CA). The gray-scale levels and curves for Figures 2, 3, and 8 were adjusted slightly in Photoshop in order to optimize the fidelity of the electron micrograph images for publication and reproduction. The RGB color values for the surface models are as follows: presynaptic membrane (200,200,225); synaptic vesicles (125,125, 255); ribs (255,197,31); beams (125,75,25); pins (200,100,25); spars (255,0,0); booms (100,0,150); non-AZM macromolecules (200,200,200); luminal assemblies of macromolecules (255,150,50); transmembrane segments (50,125,255).

### Computer Hardware and Software

For the analyses described in this study, both PC and Mac computers were used. PC computers were loaded with Windows Vista, Java(TM) 6 Update 38, IDL (7.0), EM3D Version 1.3 (IDL) and EM3D 2.0 (32 bit C^++^). Mac computers were loaded with (OS 10.6), Java (32 bit), IDL (7.0.6), EM3D (IDL) and EM3D 2.0 (C^++^).
